# Enhancement of apixaban's solubility and dissolution rate by inclusion complex (β-cyclodextrin and hydroxypropyl β-cyclodextrin) and computational calculation of their inclusion complexes

**DOI:** 10.5599/admet.1885

**Published:** 2023-08-10

**Authors:** Zainab N. Salman, Israa Al-Ani, Khaldun M. Al Azzam, Bashar J. M. Majeed, Hassan H. Abdallah, El-Sayed Negim

**Affiliations:** 1 Pharmacological and Diagnostic Research Center (PDRC), Faculty of Pharmacy, Al-Ahliyya Amman University, Amman 19328, Jordan; 2 Chemistry Department, College of Education, Salahaddin University-Erbil, Iraq; 3 School of Materials Science and Green Technologies, Kazakh-British Technical University, St. Tole bi, 59, Almaty 050000, Kazakhstan; 4 School of Petroleum Engineering, Satbayev University, 22 Satpayev Street, Almaty 050013, Kazakhstan

**Keywords:** apixaban, HPβCD, βCD, capsule, bioavailability, solubility

## Abstract

**Background and Purpose:**

Apixaban (AP) is a factor X inhibitor, an orally active drug that inhibits blood coagulation for better prevention of venous thromboembolism. It has poor solubility, dissolution rate and low bioavailability. The aim of this study was to improve the aqueous solubility and dissolution rate of oral AP as a step to enhance its bioavailability by preparing it as an inclusion complex with beta- and hydroxy propyl beta-cyclodextrin.

**Experimental Approach:**

A simple, rapid method of analysis of AP was developed using ultraviolet spectrophotometry (UV) and partially validated in terms of linearity, precision and accuracy, recovery, and robustness. AP was prepared as a complex with beta cyclodextrin (βCD) and hydroxy propyl beta cyclodextrin (HPβCD) in weight ratios 1:1, 1:2, and 1:3 by kneading, solvent evaporation and spray drying methods and characterized by Fourier transfer infra-red (FTIR), differential scanning calorimetry (DSC), and percent drug content in each of the prepared complex. Using the computer simulation, the interactions of AP with βCD and HPβCD were investigated.

**Key Results:**

The phase solubility study showed that the solubility of AP was greatly enhanced from 54×10^-3^ mmol /L to 66 mmol/L using HPβCD with acceptable stability constant. Computer docking supports the formation of a stable 1:1 complex between AP and CD’s. The dissolution test results showed that the complex gave a significantly higher percentage of drug release (95%) over one hour compared to the free AP (60%) (p<0.05).

**Conclusion:**

AP- HPβCD complex in the ratio of 1:2 (w/w) can significantly improve the solubility and in vitro dissolution rate of AP.

## Introduction

The problem of low solubility is a major challenge in the formulation of new drugs [1]. Poor solubility might lead to slow and incomplete absorption and low bioavailability [2,3]. The enhancement of drug solubility, thereby enhancing its oral bioavailability, remains one of the most challenging steps of the drug development process, especially for oral administrated drug delivery systems.

Many techniques can be utilized for solubility enhancement, especially those of Class II and IV BCS (Biopharmaceutical Classification System) drugs, to improve their oral bioavailability. Techniques include co-solvents, micronization, changing in the dielectric constant of the solvent, use of amorphous forms, chemical modification of the molecule, use of surfactants, solid dispersion techniques, eutectic mixtures, inclusion complex, manipulation of pH of solvent, utilization of hydrates or solvates, utilization of soluble prodrugs, application of ultrasonic waves, functional polymer technology, and some others. Novel drug delivery technologies used in recent years for solubility enhancement of insoluble drugs are size reduction technologies, lipid-based delivery systems, micellar technologies, and porous microparticle technology. The selection of which method would be based on certain criteria such as properties of a drug, nature of excipients to be chosen, and nature of the desired dosage form as well as cost and industrial issues [4-6].

Apixaban (AP) was developed by Bristol-Myers Squibb using the trade name of “Eliquis”, with high potency and selectivity, and is a very effective inhibitor for the blood coagulation factor *X*_a_ [7]. It is an orally developed drug that inhibits blood coagulation for better prevention of venous thromboembolism (VTE) after hip or knee replacement surgery as an alternative to previously used warfarin [8,9]. It is also indicated for the prevention and treatment of other thromboembolic diseases.

**Figure 1.**
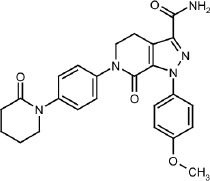
The chemical structure of AP.

AP is {1-(4-methoxyphenyl)-7-oxo-2)-4]-6-oxopiperidin-1-yl)phenyl]-5,4-dihydropyrazolo-[3,4-c]-pyridine-3-carboxamide}, its chemical structure is shown in Figure 1 [10]. AP has a molecular weight of 459.5 g/mol, and the chemical formula of C_25_H_25_N_5_O_4_. It is a white to pale yellow powder with a melting point equal to 326 °C. It has good solubility in dimethyl sulfoxide and dimethylformamide but low water solubility of (0.028 mg/mL at 24 °C). Different references give different solubility values for AP [11].

AP is absorbed along the gastrointestinal tract GIT with the distal part and descending colon contributes up to 55 % of total absorption. It also shows dissolution limited absorption, which also results in slow absorption, and so, *C*_max_ is achieved in 3-5 hours, while in rats, absorption was found to be faster and *C*_max_ was achieved in 1-2 hours [12]. Evidence shows that AP is a p-gp substrate and its efflux is affected by p-gp inhibitors, like ketoconazole or cyclosporin on the Caco-cell membrane [13]. AP mainly distributes extracellular fluids with a volume of distribution of 21 L and is 87 to 90 % bound to plasma protein, mainly albumin [14]. The half-life of AP is about 12 hours [15] and the elimination of AP involves metabolism, biliary excretion, and renal excretion of unchanged drug. The pathways of metabolizing AP include O-demethylation, hydroxylation, and sulfation of hydroxylated *O*-demethyl AP with a primary metabolism going on through cytochrome P450 (CYP) 3A4, with slight help from CYP2C9, CYP2C19, CYP2C8, and CYP1A2. A similar profile was noticed in rats in the preclinical study phase; the O-dimethyl AP was the major metabolite [16].

Cyclodextrins are cyclic oligomers capable of producing water-soluble inclusion complexes with small molecules and portions of large compounds. These biocompatible, cyclic oligosaccharides will not cause an immune reaction and have little toxicity in animals and humans. Cyclodextrins have multiple purposes in the pharmaceutical industry, including increasing the bioavailability of drugs. Cyclodextrin-containing polymers are studied and their use in drug delivery presented an interest in the use of cyclodextrin-containing polymers to provide a better ability in the delivery of nucleic acids [17].

The aim of this study is to prepare and evaluate βCD-AP and HPβCD-AP inclusion complexes to improve the solubility and dissolution rate of an oral dosage form of AP.

## Experimental

### Chemicals and instrumentation

Apixaban (purity 99.5 /Sigma), βCD and HPβCD /Sigma) A Hitachi Ratio Beam U-1800 spectrometer, EVISA, Ependorff 5810 centrifuge with a maximum rpm of 11,000 (NY, USA), Vortex Mixer (Labinco L46, Netherland), Eppendorf centrifuge 5810 R, Balance Mettler 96 (AT300, Sartorius, five decimals), pH meter (Sartorius 7110), Sonicator (Elmasonic S100), water bath BM100 with digital thermostat, IR-Prestige-21 FTIR spectrometer (Shimadzu, Japan), Buchi Nano Spray Dryer B-90 HP were used throughout the study.

### Development of analytical method based on UV absorbance

AP is slightly soluble in water. Absolute ethanol and methanol were tried in this study to prepare AP stock solution. The best solubility was obtained in methanol (1 mg/mL). Good solubility was also obtained from ethanol (0.88 mg/mL) that covered the required and expected concentrations. Due to methanol toxicity and methanol residue avoidance, ethanol was chosen as a solvent in addition to ethanol–water mixture for more dilute samples. The stock solution of AP in ethanol was prepared by dissolving 25 mg AP in 50 mL ethanol with gentle stirring and then filtration to get a clear solution. Dilution to 50 and 100 μg/mL were prepared using ethanol and scanned from 200-400 nm in UV spectrophotometer (model U-2000, Hitachi, Tokyo, Japan). Quartz cuvette was used and ethanol as blank and control. βCD and HPβCD soluble in distilled water were also scanned between 200-400 nm to ensure the specificity of the method when used to measure AP in the prepared complexes.

### Method validation

Different dilutions of AP were made from the stock solution in ethanol and used in different tests of validation according to the ICH guideline of pharmaceutical analysis. Linearity, precision and accuracy, recovery and robustness were performed to validate the developed method.

Calibration concentrations used in the linearity test were 15, 25, 30, 50, 60, 70, 80 and 90 μg/mL. Although 5 μg/mL was also tested for linearity as a lower limit of quantification. Absorbance was read trice and average ± SD was recorded at 278 nm.

Inter and intra-day precision of the study were examined and established by analysis of 6 samples (calibration concentrations) with three replicates. Within-run accuracy and precision of the method were calculated by analyzing six samples with three replicates. Relative standard deviation (RSD) was measured using the proportions of the standard deviation to the average. The comparison of practical amounts from the controls with actual values present in the samples provides the accuracy of this method.

Recovery is a ratio of the concentration of analyte available in or mixed with the analytical part of the test substance. This would be extracted and submitted for measurement (ICH guidelines). The test powder and capsule were used to test the recovery of the method. The formula contained AP beside other excipients prepared to compare the dissolution rate of the capsule containing the complex to the mixture of drug and excipients. One capsule contains 5 mg AP with the powder mix. A powder weight containing 1 mg AP in a powder blend was taken and dissolved in 5 mL ethanol, filtered through membrane filter 0.45 and 2 mL was diluted with ethanol to 10 mL to get theoretically 20 μg/mL solution. Three solutions were prepared from 3 capsules and measured spectrophotometrically at 278 nm. The other two concentrations (*c*) were prepared, 50 and 100 μg/mL, following the same procedure. Each time drug recovery was calculated by equation (1), in addition to RSD as a precision parameter:


(1)

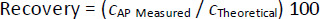



Robustness studies the effect of variation in conditions on the result. Different conditions were chosen for measuring a sample of 30 μg/mL AP. Conditions such as changing the wavelength ± 5 nm and warming of sample test to 30 °C and changing the instruments from other laboratories were used (Medicinal Chemistry lab in AAU). Other solvent was used (methanol). Each sample was read in triplicate and accuracy and RSD were calculated.

### Preparation of AP-βCD and AP-HPβCD inclusion complexes

All the following methods were applied on βCD and HPβCD in the same way and 3 different ratios (1:1, 1:2, and 1:3 w/w).

### Kneading method

In the kneading method, HPβCD or βCD was weighed (25 mg) and put in a mortar, then mixed with enough amount of water-ethanol mixture to get a thick paste. To this paste, AP was gradually incorporated in a total of up to (25 mg). The kneading process was continued for 1 hour manually in one direction. Later, enough solvent was mixed to have a consistent paste, and then it was dried at 40 °C for 48 hours in (Memert oven). Then, the dried mixture was crushed gently using the mortar and pestle. The scratched mixture was sieved using a mesh sieve (#65). Then, the formed complex was stored in a closed container. The above procedure was repeated to make the weight ratios of 1:1, 1:2, 1:3 for both βCD and HPβCD.

### Solvent evaporation method

In this method, 25 mg AP was dissolved in 50 mL ethanol. The specified amount of βCD or HPβCD was dissolved in 25 mL of distilled water. The above-prepared solutions were mixed with the help of a magnetic stirrer, and then the mixture was stirred for 8 hours. Later, most of the solvent was removed using a rotary evaporator (heating at 50 °C) at constant stirring. Reduced pressure was applied to remove the residual solvent from the mixture. The final product was placed in an oven at 40 °C for 48 hours to eliminate the excess solvent. The mixture was scratched and sieved using a mesh sieve (#65) and then stored in a tightly closed container. The above procedure was repeated to make the ratios of 1:1, 1:2, 1:3 for both βCD and HPβCD.

### Spray drying method

Buchi Nano Spray Dryer B-90 HP was used to perform the spray drying method. AP was dissolved in ethanol (50 mL), then HPβCD or βCD was dissolved in distilled water (50 mL). The two mixtures were combined by sonication for 20 minutes to provide a clear final solution. Later, the solution was undergone for spray dry process, and the drying conditions were as follows: Temperature (outlet); 90 °C, temperature (inlet); 168 °C, flow rate (solution) 1000 mL/h; flow rate (air) 400 mL/h. The final inclusion complex was crushed and sieved using sieve mesh size #65. After sieving, the inclusion complex was stored in a tight container. The above procedure was repeated to make the ratios (1:1, 1:2, 1:3) and for both βCD and HPβCD.

### Characterization of the prepared complex

After the preparation of the eighteen samples of AP-CD and AP- HPβCD complexes, they were characterized by the following methods:

#### Fourier transform infrared spectroscopy (FTIR)

FTIR was used as a characterization technique for the formation of complexes. AP, βCD and HPβCD, and the prepared complexes were scanned separately to detect the changes and identify peaks (only a representative chart is included).

#### Differential scanning calorimetry (DSC)

DSC was performed on selected samples for further confirmation. DSC (Model DT-60, Shimadzu) was used to perform this test. AP, AP-βCD, and the AP-HPβCD complex were scanned from 0 – 360 ℃ at 10 ℃/min. Thermograms were recorded and examined.

### Percent yield of the prepared complex

The yield (%) of each prepared complex was calculated as follows, equation (2):


(2)

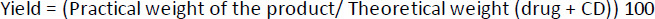



### Percent drug content in the prepared complex

This test will give the efficiency of the complexation process. It also reflects the efficiency of the method. For all types of complexes prepared, the percent AP in the complex was determined by dissolving an amount of complex equivalent to a specified amount of AP (25 mg) in 50 mL distilled water and shaking for 1 hour. Several amounts were tried that gave a clear solution. Then 50 mL ethanol was added and stirred for 2 hours to dissolve AP. Suitable dilutions were made after filtration and then read spectrophotometrically at 278 nm.

### Phase solubility study

A solubility study was conducted in distilled water with the Higuchi and Connors method [18]. Different amounts of HPβCD (depending on the results of characterization) were dissolved in 20 mL distilled water and the excess amount of AP was added. Samples were placed in a water bath at 25 °C for 24 hours with agitation at a rate of 50 rpm until equilibrium was achieved. Samples of 3 mL were withdrawn and filtered through a 0.45 μm membrane filter. Filtrate (100 μL) was diluted appropriately and assayed at 278 nm. The concentrations used are given in Table 1.

The solubility of pure AP was also tested using distilled water and an excess of AP in the same conditions. A phase solubility graph was then constructed, and the apparent stability constants (*K*_st_) were determined from the phase solubility analysis graph using equation (3):


(3)

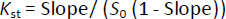



where *K*_st_ is the stability constant of the complex, *S*_0_ is the absolute solubility of AP in the absence of CDs [18].

### Computer simulation of inclusion complex

The structure of the guest molecule, AP, was downloaded as a mol file from the ChemSpider database (www.chemspider.com) [19]. The structure was optimized using the AM1 method using Gaussian09 software [20]. On the other hand, the crystal structure of the host molecule βCD was downloaded from Cambridge Crystallographic Data Centre (CCDC) under the reference number. The structure was checked, and the crystalized molecules were removed. In addition, hydroxypropyl groups were added using GaussView [20] to produce the hydroxypropyl-βCD structure. The optimized structure of the guest molecule was then docked at the center of the host molecules, βCD and HPβCD. To prepare the host-guest complexes for docking calculations, Kollman United Atom (KUA) charges were added in a grid box with 60x60x60 dimensions using Autogrid as part of Autodock 4 software (Molinspiration Database). All possible conformations were docked at the center of the host molecule using the Lamarckian genetic algorithm with 250 runs for each guest molecule.

### In vitro dissolution of AP-HPβCDs inclusion complexes versus free AP

Powder dissolution was performed to compare the dissolution of free AP with the complex prepared in a dissolution apparatus USP type II (paddle type). The dissolution medium consists of 900 mL (pH 6.8 phosphate buffer) maintained at 37±1 °C, with paddle rotation at the speed of 50 rpm. Three jars were used for free drug and 3 jars for the complex. Five mg AP free was added in each jar specified for “free drug” and an amount of the dry complex powder equivalent to 5 mg AP was added to the jars specified to the “complex powder” and labeled. Samples of 5 mL dissolution media were withdrawn at various time points and replaced with fresh buffer at time points: 5, 10, 20, 30, 45, and 60 minutes. Samples were analyzed using the validated method and the percent cumulative AP release versus time point were calculated and plotted.

### Preparation of AP capsules

AP is found in the market as tablets contain 2.5 or 5 mg AP and (anhydrous lactose (bulking agent)), microcrystalline cellulose (bulking agent), croscarmellose sodium (disintegrant), sodium lauryl sulfate (surfactant), and magnesium stearate (lubricant) [21]. Formulation of tablets and their parameters was left as future work. A simple capsule dosage form was prepared to compare a formulation and perform drug release on a dosage form.

A simulated capsule containing 5 mg AP, 94 mg Avicel PH102, and 1 mg SLS in each capsule was prepared. No disintegrant or lubricant was needed. Each test capsule contained an amount of complex equivalent to 5 mg AP, 1 mg SLS and the weight was completed to 100 mg by Avicel. A small batch of 20 capsules was prepared by mixing ingredients and filling in a lab-scale capsule filling machine. To ensure uniformity of weight, capsules were weighed individually and any capsule deviating by 5% and more from 100 mg was excluded.

### Dissolution test of AP capsules

The dissolution test was performed on the prepared AP capsule and the complex-containing capsules in the same conditions as the powder dissolution. Each sample withdrawn was filtered through a nylon membrane filter (0.45 μm), diluted by methanol (blank) and analyzed using UV spectrophotometer at 278 nm. The percent cumulative of AP release of each experiment were calculated and plotted. To investigate the kinetic of drug release, several release models were applied. These models are shown in Table 2. The aim is to get a straight line, and the highest correlation (*R*^2^) would be considered the closest model. All models were fitted using Microsoft Excel.

### Statistical analysis

Statistical analysis was used in this study. For the method of analysis, means, standard deviations (SD), coefficient of variation (CV) and their percentages were all used. Determination of correlation coefficient (*R*^2^) was also used. In terms of dissolution and phase solubility analysis study, a *t*-test was used to compare the target parameters of both drugs when given alone and in combination to test if any significant difference in CI 5% is present. The online “GraphPad” or Excel software program was used for this purpose.

## Results and discussion

### Method development and validation

**Figure 2.**
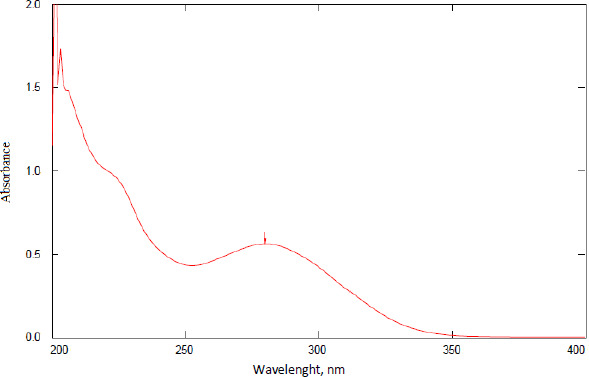
UV scanning of a solution containing 50 μg/mL AP in ethanol. The wavelength of 278 nm exhibited the highest absorption.

A sample of AP was scanned for the determination of the *λ*_max_. Figure 2 shows the scanning result of a solution containing 50 μg/mL AP in ethanol. The wavelength of 278 nm gave the maximum absorbance. Scanning of ethanol alone and different concentrations of βCD and HPβCD was also performed to ensure the specificity of the *λ*_max_ chosen. These results proved that the solvent, βCD, and HPβCD have negligible absorbances at the selected *λ*_max_ of AP, which is 278 nm (data are not shown). This *λ*_max_ was selected to continue validation and other measurements in the work.

### Method validation

The method validation followed the ICH guidelines in terms of linearity, precision, accuracy, recovery, and robustness.

**Figure 3.**
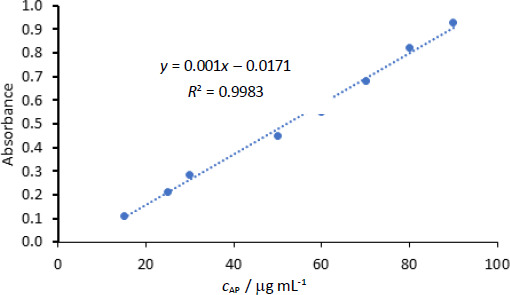
The linearity plot of AP shows the linear regression equation and correlation coefficient.

Linear regression was observed in the concentrations range of 15-90 μg/mL. The correlation coefficient for the linearity test was 0.998. Figure 3 shows the linearity plot of AP. All tests used in the measurement of AP had the target of dilutions to produce concentrations within this range of 15-90 μg/mL to ensure linearity of readings versus concentrations. The back-calculation gave RSD 0.24-2.66 %.

The precision of an analytical procedure expresses the closeness of agreement (degree of scatter) between a series of measurements obtained from multiple sampling of the same homogeneous sample under the prescribed condition. Precision is measured by the RSD of a series of readings which should not exceed 2.0 % according to the ICH guidelines. While accuracy is expressed in “percent” measured concentration relative to the theoretical concentration.

Measurements were made over 2 days. Table 3 shows measurements at day 1 (interday precision) and day 2 (intraday precision) of AP. The RSD for all readings ranged 0.88 to 1.2 %, which complies with the ICH. The accuracy or recovery of AP from the samples ranged between 96.4 to 103.2 %, knowing that 90 to 110 % is accepted as per the ICH guidelines.

The powder mixture of the test capsule of AP was used to test the recovery of the method. The formula contained AP beside other excipients prepared to compare the dissolution rate of the capsule containing the complex to the mixture of drug and excipients. The recovery or accuracy of the previous test was calculated on the pure AP dissolved in ethanol samples. While this test examines the ability of the method to quantify AP in the powder mixture of the formula. This necessitates high specificity to the compound and negligible effect of excipients. A good method would recover the amount of API in higher or lower concentrations using the same conditions with high accuracy.

Table 4 shows the results of the recovery test of AP, showing the accuracy of measurement and RSD as a measurement of precision. Results gave percent recovery of AP from all formulas 97.3-98.2% with precision between 0.9 and 1, which fulfills the criteria of ICH guidelines.

Robustness studies the effect of variation in conditions on the result and tests how much the method is resistant to these changes. Different conditions were chosen for measuring a sample of 50 μg/mL AP. The accuracy of measurement of AP ranged between 97.3 to 99.8 %, which indicated high resistance of the method to changes in the condition specified, as shown in Table 5.

The wavelength was changed from 278-275 nm one time to 285 another time to test the possibility of a result change. Two instruments were used, and the temperature of the test solution was elevated to 30 °C as well as blank. These parameters showed a very slight difference in the accuracy of the measured concentration tested. In conclusion, the method successfully handled some changes in conditions within the ICH guidelines' requirements.

### Characterization of the prepared complex

The prepared complexes by the three mentioned methods were evaluated to confirm complex formation, percent yield and AP content in each powder complex prepared. Based on the results, the method of preparation was evaluated, and the best complex was chosen for further work.

**Figure 4.**
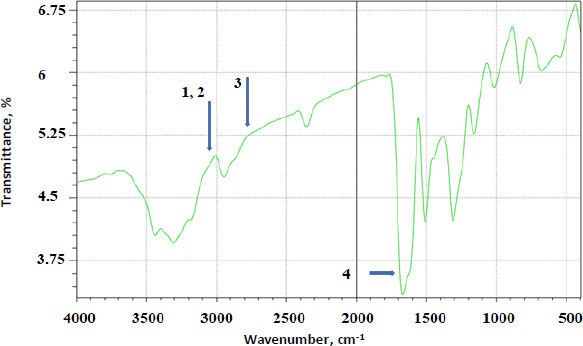
FTIR spectrum of AP showing the major peaks (1) 3325.0 cm^-1^ (N-H stretching of sulphonamido gr.), (2) 3300-3500 cm^-1^ (N-H stretching of amido gr.) (3) 2930 cm^-1^ (CH stretching of CH_2_) and (4) characteristic C=O of amide at 1628.9 cm^-1^.

**Figure 5.**
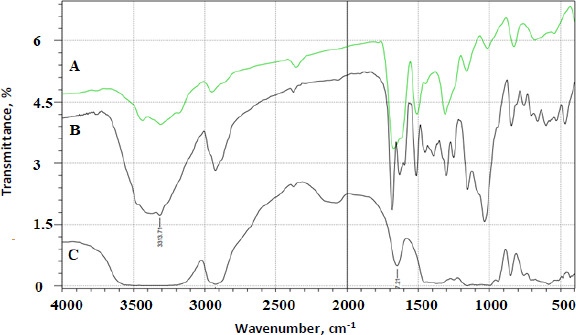
Overlay spectra of AP (A), AP-βCD (B), and AP-HPβCD (C). The most characteristic peaks in the two complexes AP-βCD (B), and AP-HPβCD (C) appeared with less intensity of the C=O amide group of AP, which might be attributed to the hidden nature of this group inside the cone of the CDs.

FTIR analysis was done on all the prepared complexes. Below are a few examples of the prepared complexes. Figure 4 shows the FTIR spectrum of AP. Figure 5 shows the FTIR overlay spectra of AP (A), AP-βCD (B), and AP-HPβCD (C) results.

AP identifying peaks observed were: (1) 3325.0 cm^-1^ (N-H stretching of sulphonamido group), (2) 3300 to 3500 cm^-1^ (N-H stretching of amido group) (3) 2930 cm^-1^ (CH stretching of CH_2_) and (4) characteristic C=O of amide at 1628.9 cm^-1^.

The prominent peaks for βCD alone are 3398 cm^-1^ (O-H), 2930 cm^-1^ (C-H stretching), 1628 to 1598 cm^-1^ (H-O-H bending), 1156 cm^-1^ (C-O stretching), 1028 cm^-1^ (C-O-C stretching). The IR-abundant peaks for HPβCD alone are 3395 cm^-1^ (O-H), 2930 cm^-1^ (C-H stretching), 1628 cm^-1^ (H-O-H bending), 1156 cm^-1^ (C-O stretching), 1032 cm^-1^ (C-O-C stretching).

### Yield and content of AP-βCDs inclusion complexes

AP-βCD and HPβCD complexes were prepared on a laboratory scale. However, percentage yield was important to determine how efficient the method and the skills used were. The percent yield of each prepared complex was calculated, as shown in Table 6. All the methods used were under good control and gave a very high percent yield (89 – 99 %). Spray drying gave the highest yield with minimum loss.

Measuring the content of AP included in each type of the complex formed generally gave a high loading efficiency. Two variables can be explained: the weight ratio of AP to CD and the method of preparation. Results in Table 6 showed that the kneading method gave the highest loading efficiency of AP in the complex for both βCD and HPβCD. Solvent evaporation and spray drying methods gave a bit less content drug inclusion. This difference is attributed to the method of preparation and how the molecule gets inside the CD cone. Results also showed that increasing the weight ratio of AP from 1:1 to 1:2, both with βCD and HPβCD, respectively, increased the amount of drug loading, but a further increase in the CD ratio did not give a noticeable increase in the amount of drug loading. For that reason, AP-βCD 1:2 and AP-HPβCD 1:2 was chosen for further studies because there was no need to increase CD concentration in the formula if there was no prominent impact on the solubility of AP.

### Characterization of AP-βCD (1:2) and AP-HPβCD (1:2) complex by DSC

**Figure 6.**
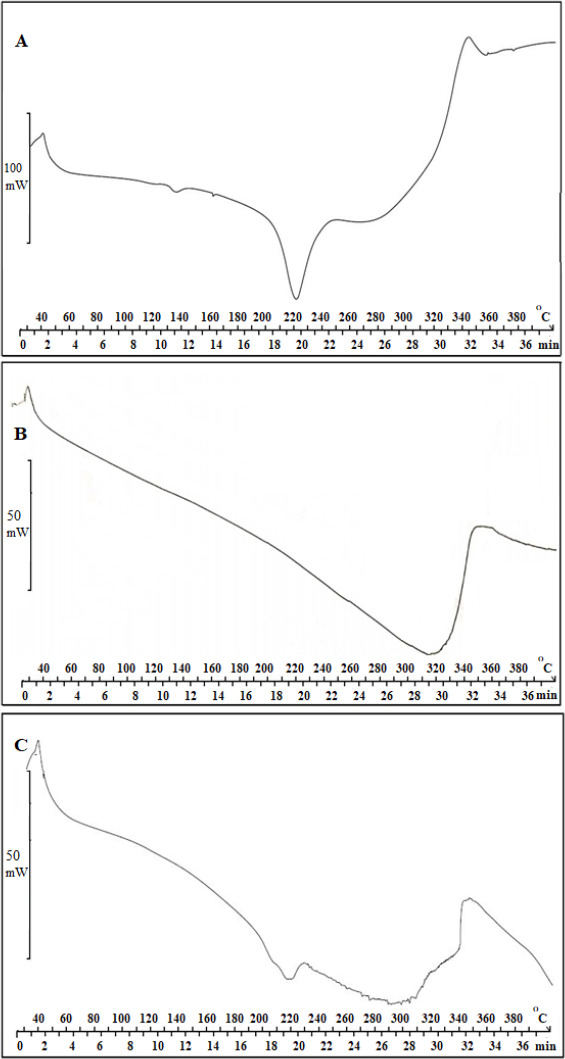
DSC thermograms of AP (A), HPβCD (B) and AP-HPβCD complex ratio (AP:HPβCD) (1:2 w/w) (C).

Further characterization of AP-HPβCD (1:2) complex was done by DSC for further confirmation. Results are shown in Figure 6 A (AP), -B (HPβCD), and -C (AP-HPβCD 1:2 complex), which was chosen for the formulation of the capsules. Results show the melting point of AP of approximately 220 °C from DSC. Figure 6 B also shows the DCS thermogram of HPβCD showing a broad peak in the range 280 – 340 °C. The complex thermogram shows that the sharp peak of AP is embedded in the HPβCD and is not clearly distinguished, indicating the formation of an inclusion complex between AP and HPβCD (C).

### Phase solubility analysis of AP-βCD and AP-HPβCD inclusion complexes

**Figure 7.**
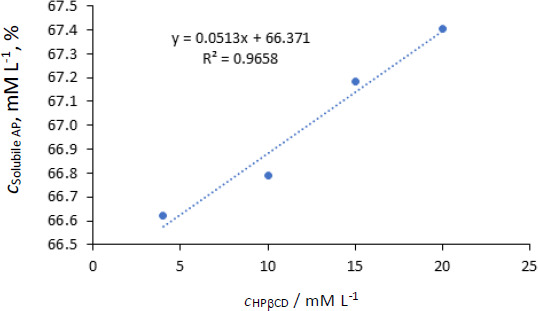
Phase solubility diagram of AP with HPβCD.

The phase solubility profile of AP-HPβCD is presented in Figure 7. The solubility of pure AP was calculated approximately as 54.4x10^-3^ mmol (25 μg/mL) (28 μg/mL reported by [22]). Using suitable dilutions, concentrations, mol/L of solubilized AP by different concentrations of HPβCD were calculated and given in Table 7.

From Figure 7, the solubility of AP increased proportionally with an increase in the concentration of HPβCD (R^2^ = 0.97). However, the increase of molar concentration of HPβCD from 4-20 (5 folds) resulted in a very slight increase in the solubility of AP. 1:1 relation means that every molecule of the host compound incorporates one molecule of the guest (AP).

The lowest concentration of HPβCD used (4 mM) resulted in 1224 fold increase in the solubility of AP in water (from 54.4 μM to 66.622 mM). Further increase in HPβCD resulted in a slight increase in solubility. However, a straight line with a good correlation was obtained in Figure 7, and the stability constant was calculated by equations (3) and (4):


(3)

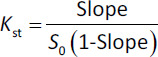




(4)

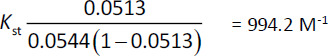



The stability constant (*K*_st_) equals 994.2 M^-1^. It was reported that *K*_s_ value between 50 and 5000 M^−1^ was considered the most suitable for the improvement of solubility and stability of poorly soluble drugs [23]. In conclusion, HPβCD increased the solubility of AP significantly with good stability of the complex in a ratio 1:1 host-guest molecules.

### Computer calculations

Docking calculations offer essential information regarding the conformation of the guest-host complexes and produce a quantitative parameter of the binding affinity of different complexes. AP has shown the highest binding affinity in complexing with HPβCD (-7.62 kcal/mol) followed by βCD (-6.81 kcal/mol), where it agrees with the experimental part attained.

**Figure 8.**
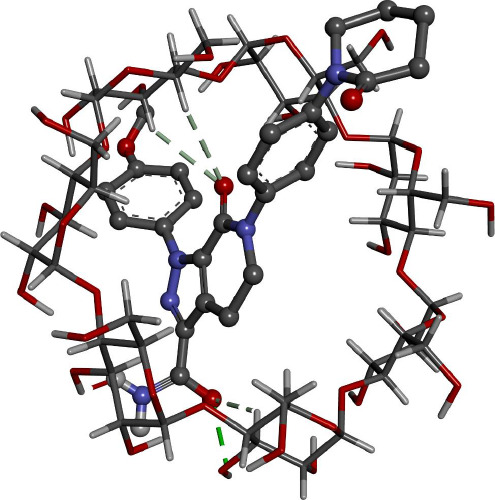
Interaction of AP with βCD as proposed by Gaussian09 software.

**Figure 9.**
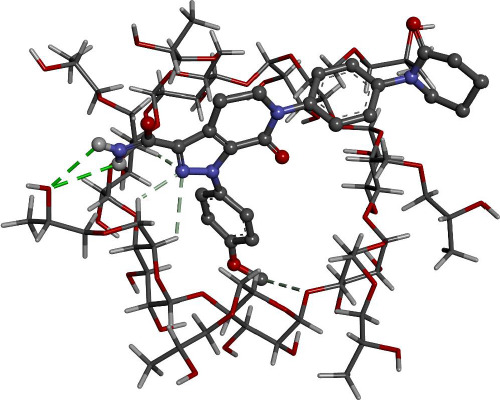
Interaction of AP with HPβCD as proposed by Gaussian09 software.

Figures 8 and 9 give better insight into the binding interaction between the host-guest complexes. In both cases, the amide group is included inside the cone. The same result was obtained from the FTIR analysis, where the amide peak was shortened, and its absorption was weakened.

### Dissolution studies

**Figure 10.**
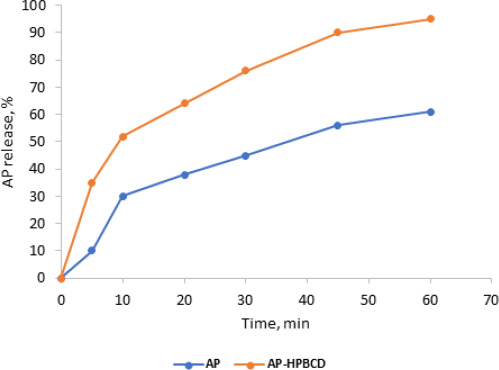
Dissolution profile of AP free powder and AP-HPβCD (1:2) powder at 37 °C, 50 rpm, USP dissolution apparatus type II.

**Figure 11.**
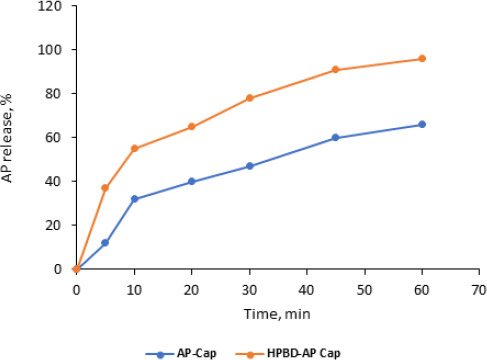
Dissolution profile of AP free AP-HPβCD (1:2) from capsule dosage form 37 °C, 50 rpm, USP dissolution apparatus type II.

The dissolution rate profiles of standard AP alone and AP-HPβCD (1:2) system in pH 6.8 at 37.0 ± 0.5 °C are displayed in Figures 10 and 11. The release rate profiles were expressed as the amount (amount released×100 / total amount (5 mg)) of AP released from the complexes against time in minutes.

Figure 10 shows an improvement in the dissolution rate of AP released from AP-HPβCD (1:2) powder over AP pure powder. This indicates improvement of solubility of AP by complexation with HPβCD. The amount released in 10 minutes (*t*_10_) was taken as a comparison point and results showed a significant increase in AP dissolved from HPβCD complexes (52 ± 3.5) 5 compared to AP pure powder (30 ± 4.0) with (*p*<0.05).

AP release from capsule dosage form (free AP versus complex) is shown in Figure 11. Time started after the capsules were perforated in the media and the powder started to spread in the media in about 20 ±3 minutes.

Results showed the dissolution was significantly improved compared to the free-AP capsules. T_10_ from capsules containing the complex was 55±3.4 % compared to 32±1.5 %. These results are close to those of free powder.

### Kinetics of drug release

The *in vitro* drug release mechanism of AP from capsules in buffer pH 6.8 at 37.0±0.5 °C can be described by fitting the dissolution data in five different kinetic models such as zero-order, first-order, Peppas, Higuchi, and Hixson–Crowell (Table 8). The linearity was calculated to determine the best-fit model and possible mechanism of drug release from the formula.

Drug release kinetics was studied for the capsules (reference and AP-HPβCD (1:2) containing capsules) using the models shown in Table 8 using Microsoft Excel for the model fitting and calculations. The aim is to get a straight line that explains the linear relation between the specified variables. Linearity is evaluated by correlation coefficient (R^2^) to choose the best-fit model (Table 8). The equation that best suits the dissolution data where the values of *R*^2^ were >0.9.

Results of model fitting (Table 8) showed that AP is released from the complex in a capsule dosage form following “first order” release kinetic with a total amount released in 1 hour of more than 90 %, which is a suitable behavior for immediate release (IR) dosage form indicating first-order release mechanism. These results also indicated that the complex significantly improved the drug release and dissolution behavior.

## Conclusions

AP forms a complex with βCD and HPβCD with good loading efficiency using the kneading method, solvent evaporation method and spray drying method. The highest percentage was gained from the kneading method in a ratio of 1:2 AP-HPβCD. Also, the phase solubility study showed that AP reacts with HPβCD in a ratio of 1:1 stoichiometry with a stability constant equal to 994 M^-1^.^.^ Computer simulation of the AP-CD interaction supports the formation of 1:1 complex. For characterization purposes, FTIR and DSC showed a physical interaction between AP and HPβCD. Additionally, drug dissolution was significantly higher from the powder of the complex and the formula of gelatin capsules compared to free AP and AP in capsules without the complex.

## Figures and Tables

**Table 1.** Concentrations of AP (using excess amount) and HPβCD used in phase solubility study.

Sample code	*c*_HPβCD_ / mmol L^-1^
Sample 1	4.00
Sample 2	10.0
Sample 3	15.0
Sample 4	20.0

**Table 2.** Kinetic of drug release models tested.

Model Name	Y-axis	X-axis
Zero-Order	Cumulative amount of drug release	Time
First order	log (Cumulative amount of drug remained)	Time
Peppas model	log (Cumulative amount of drug release)	log (Time)
Higuchi model	Cumulative amount of drug release	Time^1/2^
Hixon Crowel model	*W*_0_^1/3^ – *W*_t_^1/3^*	Time

*W_0_ is initial amount, *W*_t_ is amount remaining

**Table 3.** Results of Inter and Intra-day precision and accuracy of AP.

Sample ID	*c*_Theoretical_ / μg mL^-1^	Absorbance (average of *n*=3)	*c*_Measured_ / μg mL^-1^	Accuracy, %	RSD, %
Day 1					
Sample1	50	0.471	49.28	98.6	1.2
Sample2	50	0.473	49.47	98.9	
Sample3	50	0.459	48.17	96.4	
Sample4	50	0.480	50.12	100.2	
Sample5	50	0.472	49.37	98.8	
Sample6	50	0.470	49.19	98.4	
Day 2					
Sample1	50	0.496	51.60	103.2	0.88
Sample2	50	0.481	50.21	100.4	
Sample3	50	0.469	49.10	98.2	
Sample4	50	0.488	50.86	101.7	
Sample5	50	0.476	49.75	99.5	
Sample6	50	0.471	49.28	98.6	

**Table 4.** Results recovery test of AP from capsule dosage form showing accuracy and precision AP in test formulas.

Sample ID	*c*_Theoretical_ / μg mL^-1^	Measured absorbance	*c*_Measured_ / μg mL^-1^ (from formula)	Accuracy, %	Average accuracy, %	RSD, % (Precision)
Sample1-1	40.0	0.359900	39.00	97.5	97.9	0.9
Sample1-2	40.0	0.362060	39.20	98.0		
Sample1-3	40.0	0.362924	39.28	98.2		
Sample2-1	60.0	0.570500	58.50	97.5	98.2	1.0
Sample2-2	60.0	0.574388	58.86	98.1		
Sample2-3	60.0	0.580220	59.40	99.0		
Sample3-1	90.0	0.900980	89.10	99.0	97.3	0.97
Sample3-2	90.0	0.881540	87.30	97.0		
Sample3-3	90.0	0.879596	87.12	9.86		

**Table 5.** Results of robustness of method AP (50 μg mL^-1^) test showing different conditions used, accuracy and precision.

Sample ID	Absorbance	*c*/ μg mL^-1^		Accuracy, %	RSD, %
		Theoretical	Measured (*n*=3)		
Robustness + 5 nm (285 nm)	0.693	50	49.86	99.8	0.6
Robustness – 5 nm (275 nm)	0.691	50	49.72	99.6	0.9
Robustness-instrument 1	0.687	50	49.37	99.1	0.58
Robustness-instrument 2	0.674	50	48.11	97.3	1.2
Robustness 30℃	0.690	50	48.78	98.4	0.7
AP in methanol		50	49.1	98.2	0.9

**Table 6.** Yield and content of AP-βCD and AP-HPβCD complexes.

Method of preparation	Type of complex	AP:CD weight ratio	Yield, %	Loading efficiency, %
Kneading	βCD	1:1	91	90.6
Kneading	βCD	1:2	93	95.5
Kneading	βCD	1:3	94	95.6
Kneading	HPβCD	1:1	89	89.2
Kneading	HPβCD	1:2	92.5	97.2
Kneading	HPβCD	1:3	92	96.3
Solvent evaporation	βCD	1:1	96	88
Solvent evaporation	βCD	1:2	98	93
Solvent evaporation	βCD	1:3	98	92
Solvent evaporation	HPβCD	1:1	95	85
Solvent evaporation	HPβCD	1:2	96	93
Solvent evaporation	HPβCD	1:3	97	92
Spray drying	βCD	1:1	99	85
Spray drying	βCD	1:2	99	92
Spray drying	βCD	1:3	99	93
Spray drying	HPβCD	1:1	99	82
Spray drying	HPβCD	1:2	99	94
Spray drying	HPβCD	1:3	99	95

**Table 7.** Results of phase solubility study.

*c* / mmol L^-1^			
HPβCD	Soluble AP	Soluble AP total - *S*_0_ +*S*_complex_	Soluble AP, by HPβCD
4.0	30.621	66.6764	66.622
10 .0	30.781	66.8434	66.789
15.0	30.871	67.1904	67.185
20.0	30.971	67.4084	67.403

**Table 8.** Results of model fitting for drug release from reference capsules and capsules contain (AP-HPβCD).

Model	Zero-order	First order	Peppas model	Higuchi model	Hixon Crowel model
AP release from the Reference capsule					
*R* ^2^	0.88	0.94	0.91	0.96	0.94
AP release from capsules contains (AP-HPβCD)					
*R* ^2^	0.80	0.99	0.98	0.97	0.95

## List of abbreviations

AP: Apixaban
βCD: Beta cyclodextrin and
FTIR: Fourier transfer infra-red
BCS: Biopharmaceutical classification system
SD: Standard deviations
*R* ^2^: Correlation coefficient
UV: Ultraviolet spectrophotometry
HPβCD: Hydroxy propyl beta cyclodextrin
DSC: Differential scanning calorimetry
VTE: Venous thromboembolism
CV: Coefficient of variation

## References

[1] Sharma A. Soni M. Kumar S. Gupta G.D.. Solubility enhancement—eminent role in poorly soluble drugs. Research Journal of Pharmacy and Technology 2 (2009) 220-224. https://rjptonline.org/AbstractView.aspx?PID=2009-2-2-46

[2] Hodgson J.. ADMET—turning chemicals into drugs. Nature Biotechnology 19 (2001) 722-726. https://doi.org/10.1038/90761 10.1038/90761 11479558

[3] Savjani K.T. Gajjar A.K. Savjani J.K.. Drug Solubility: Importance and Enhancement Techniques. International Scholary Research Notices 2012 (2012) 1–10. https://doi.org/10.5402/2012/195727 10.5402/2012/195727 PMC3399483 22830056

[4] Lipiniski C. Lipinski C.J Lipinski C. Lipinski C.A. Lipinski C. Lipinski C.A. Lipinski Lipinski C.A.. Poor aqueous solubility - an industry wide problem in drug delivery. American Pharmaceutical Review 5 (2002) 82-85. https://www.scienceopen.com/document?vid=dbc999fb-0f66-463c-8166-d6576f8778d5

[5] Guo Z. Boyce C. Rhodes T. Liu L. Salituro G.M. Lee K.J. Bak A. Leung D.H.. A novel method for preparing stabilized amorphous solid dispersion drug formulations using acoustic fusion. International Journal of Pharmaceutics 592 (2021) 120026. https://doi.org/10.1016/j.ijpharm.2020.120026 10.1016/j.ijpharm.2020.120026 33137448

[6] Vemula V.R. Lagishetty V. Lingala S.. Solubility enhancement techniques. International Journal of Pharmaceutical Sciences Review and Research 5 (2010) 41-51. https://globalresearchonline.net/journalcontents/volume5issue1/article-007.pdf

[7] Pinto D.J. Orwat M.J. Koch S. Rossi K.A. Alexander R.S. Smallwood A. Wong P.C. Rendina A.R. Luettgen J.M. Knabb R.M. He K. Xin B. Wexler R.R. Lam P.Y.S.. Discovery of 1-(4-methoxyphenyl)-7-oxo-6-(4-(2-oxopiperidin-1-yl) phenyl)-4, 5, 6, 7-tetrahydro-1 H-pyrazolo [3, 4-c] pyridine-3-carboxamide (Apixaban, BMS-562247), a highly potent, selective, efficacious, and orally bioavailable inhibitor of blood coagulation factor Xa. Journal of Medicinal Chemistry 50 (2007) 5339-5356. https://doi.org/10.1021/jm070245n 10.1021/jm070245n 17914785

[8] Deeks E.D.. Apixaban. Drugs 72 (2012) 1271-1291. https://doi.org/10.2165/11209020-000000000-00000 10.2165/11209020-000000000-00000 22686618

[9] Falck-Ytter Y. Francis C.W. Johanson N.A. Curley C. Dahl O.E. Schulman S. Ortel T.L. Pauker S.G. Jr C.W.C.. Prevention of VTE in orthopedic surgery patients: antithrombotic therapy and prevention of thrombosis: American College of Chest Physicians evidence-based clinical practice guidelines. Chest 141 (2012) e278S-e325S. https://doi.org/10.1378/chest.11-2404 10.1378/chest.11-2404 22315265 PMC3278063

[10] Wong P.C. Pinto D.J. Zhang D.. D. Preclinical discovery of apixaban, a direct and orally bioavailable factor Xa inhibitor. Journal of Thrombosis and Thrombolysis 31 (2011) 478-492. https://doi.org/10.1007/s11239-011-0551-3 10.1007/s11239-011-0551-3 21318583 PMC3090580

[11] Li H. Farajtabar A. Xie Y. Li Z. Zhao H.. Apixaban (I) in several aqueous co-solvent mixtures: Solubility, solvent effect and preferential solvation. The Journal of Chemical Thermodynamics 150 (2020) 106200. https://doi.org/10.1016/j.jct.2020.106200 10.1016/j.jct.2020.106200

[12] Byon W. Garonzik S. Boyd R.A. Frost C.E.. Apixaban: a clinical pharmacokinetic and pharmacodynamic review. Clinical Pharmacokinetics 58 (2019) 1265-1279. https://doi.org/10.1007/s40262-019-00775-z 10.1007/s40262-019-00775-z 31089975 PMC6769096

[13] Zhang D. He K. Herbst J.J. Kolb J. Shou W. Wang L. Balimane P.V. Han Y.H. Gan J. Frost C.E. Humphreys W.G.. Characterization of efflux transporters involved in distribution and disposition of apixaban. Drug Metabolism and Disposition 41 (2013) 827-835. https://doi.org/10.1124/dmd.112.050260 10.1124/dmd.112.050260 23382458

[14] Jaber A. Al-Ani I. Hailat M. Daoud E. Abu-Rumman A. Zakaraya Z. Majeed B.J. Al Meanazel O. Dayyih W.A.. Esomeprazole and apixaban pharmacokinetic interactions in healthy rats. Heliyon 8 (2022) e11015. https://doi.org/10.1016/j.heliyon.2022.e11015 10.1016/j.heliyon.2022.e11015 36281394 PMC9586895

[15] Ward C. Conner G. Donnan G. Gallus A. McRae S.. Practical management of patients on apixaban: a consensus guide. Thrombosis Journal 11 (2013) 1-8. https://doi.org/10.1186/1477-9560-11-27 10.1186/1477-9560-11-27 24380488 PMC3904756

[16] Wang L. Raghavan N. He K. Luettgen J.M. Humphreys W.G. Knabb R.M. Pinto D.J. Zhang D.. Sulfation of o-demethyl apixaban: enzyme identification and species comparison. Drug Metabolism and Disposition 37 (2009) 802-808. https://doi.org/10.1124/dmd.108.025593 10.1124/dmd.108.025593 19131519

[17] Davis M.E. Brewster M.E.. Cyclodextrin-based pharmaceutics: past, present and future. Nature Reviews Drug Discovery 3 (2004) 1023-1035. https://doi.org/10.1038/nrd1576 10.1038/nrd1576 15573101

[18] Doile M.M. Fortunato K.A. Schmücker I.C. Schucko S.K. Silva M.A.S. Rodrigues P.O.. Physicochemical properties and dissolution studies of dexamethasone acetate-β-Cyclodextrin inclusion complexes produced by different methods. AAPS PharmSciTech 9 (2008) 314-322. https://doi.org/10.1208/s12249-008-9042-z 10.1208/s12249-008-9042-z 18446497 PMC2976901

[19] ChemSpider database. http://www.chemspider.com/ (Accessed date: 01/08/2023).

[20] Gaussian 09, Revision D.01 Frisch M.J. Trucks G.W. Schlegel H.B. Scuseria G.E. Robb M.A. Cheeseman J.R. Scalmani G. Barone V. Mennucci B. Petersson G.A. Nakatsuji H. Caricato M. Li X. Hratchian H.P. Izmaylov A.F. Bloino J. Zheng G. Sonnenberg J.L. Hada M. Ehara M. Toyota K. Fukuda R. Hasegawa J. Ishida M. Nakajima T. Honda Y. Kitao O. Nakai H. Vreven T. Montgomery J.A. Peralta J.E. Jr. Ogliaro F. Bearpark M. Heyd J.J. Brothers E. Kudin K.N. Staroverov V.N. Kobayashi R. Normand J. Raghavachari K. Rendell A. Burant J.C. Iyengar S.S. Tomasi J. Cossi M. Rega N. Millam J.M. Klene M. Knox J.E. Cross J.B. Bakken V. Adamo C. Jaramillo J. Gomperts R. Stratmann R.E. Yazyev O. Austin A.J. Cammi R. Pomelli C. Ochterski J.W. Martin R.L. Morokuma K. Zakrzewski V.G. Voth G.A. Salvador P. Dannenberg J.J. Dapprich S. Daniels A.D. Farkas Ö. Foresman J.B. Ortiz J.V. Cioslowski J. Fox D.J.. Gaussian, Inc., Wallingford CT, 2009. https://www.scienceopen.com/document?vid=839f33cc-9114-4a55-8f1a-3f1520324ef5

[21] Apixaban in: https://www.rxlist.com/eliquis-drug.htm (Accessed date: 20/04/2023).

[22] Chen Y. Li L. Yao J. Ma Y.Y. Chen J.M. Lu T.B.. Improving the solubility and bioavailability of apixaban via apixaban–oxalic acid cocrystal. Crystal Growth and Design 16 (2016) 2923-2930. https://doi.org/10.1021/acs.cgd.6b00266 10.1021/acs.cgd.6b00266

[23] Sip S. Gościniak A. Szulc P. Walkowiak J. Cielecka-Piontek J. J. Assisted Extraction with cyclodextrins as a way of improving the Antidiabetic activity of actinidia leaves. Pharmaceutics 14 (2022) 2473. https://doi.org/10.3390/pharmaceutics14112473 10.3390/pharmaceutics14112473 36432664 PMC9695090

